# Comparing Deep-Sea Fish Fauna between Coral and Non-Coral “Megahabitats” in the Santa Maria di Leuca Cold-Water Coral Province (Mediterranean Sea)

**DOI:** 10.1371/journal.pone.0044509

**Published:** 2012-09-24

**Authors:** Gianfranco D'Onghia, Porzia Maiorano, Roberto Carlucci, Francesca Capezzuto, Angela Carluccio, Angelo Tursi, Letizia Sion

**Affiliations:** Department of Biology, University of Bari “Aldo Moro”, CoNISMa, Bari, Italy; Université du Québec à Rimouski, Canada

## Abstract

Two experimental longline surveys were carried out in the Santa Maria di Leuca (SML) cold-water coral province (Mediterranean Sea) during May–June and September–October 2010 to investigate the effect of corals on fish assemblages. Two types of “megahabitat” characterized by the virtual absence of fishing were explored. One was characterized by complex topography including mesohabitats with carbonate mounds and corals. The other type of megahabitat, although characterized by complex topographic features, lacks carbonate mounds and corals. The fishing vessel was equipped with a 3,000 m monofilament longline with 500 hooks and snoods of 2.5 m in length. A total of 9 hauls, using about 4,500 hooks, were carried out both in the coral megahabitat and in the non-coral megahabitat during each survey. The fish *Leucoraja fullonica* and *Pteroplatytrygon violacea* represent new records for the SML coral province. The coral by-catch was only obtained in the coral megahabitat in about 55% of the stations investigated in both surveys. The total catches and the abundance indices of several species were comparable between the two habitat typologies. The species contributing most to the dissimilarity between the two megahabitat fish assemblages were *Pagellus bogaraveo*, *Galeus melastomus*, *Etmopterus spinax* and *Helicolenus dactylopterus* for density and *P. bogaraveo*, *Conger conger*, *Polyprion americanus* and *G. melastomus* for biomass. *P. bogaraveo* was exclusively collected in the coral megahabitat, whereas *C. conger*, *H. dactylopterus* and *P. americanus* were found with greater abundance in the coral than in the non-coral megahabitat. Differences in the sizes between the two megahabitats were detected in *E. spinax*, *G. melastomus*, *C. conger* and *H. dactylopterus*. Although these differences most probably related to the presence-absence of corals, both megahabitats investigated play the role of attraction-refuge for deep-sea fish fauna, confirming the important role of the whole SML coral province as a refuge area from fishing.

## Introduction

Cold-water corals (CWC), as autogenic ecosystem engineers, build 3D habitats with complex structures on the predominantly homogeneous deep-sea floor providing shelter, enhanced food supply, spawning sites and nursery areas for many associated species [1], [2], [3]–[10]. In fact, faunal abundance and diversity can be significantly greater in the coral habitats than in non coral areas [5], [11]–[14]. However, it has not been proved whether corals themselves or only their structural complexity are the attracting factors for deep-sea fauna [15]. In this respect, the knowledge on the distribution and habitat use of the mobile fauna dwelling in deep-sea coral habitats remains incomplete due to the difficulties of consistently repeating standardized sampling in such complex habitats. In addition, the various deep-sea species show different vulnerability to the used gears and different reactions to the employed video systems [5], [6]. Furthermore, some species, such as large carnivorous and scavenger species belonging to the higher trophic levels, roam a vast area searching for randomly occurring large food items and can be equally distributed between sedimentary and coral habitats [8], [10], [16]. Corals can show a very patchy distribution on the slope and reefs generally show zonation of different benthic habitats with rather gradual boundaries between them [3], [17], [18], [8], [9], [19].

The mobile fauna distributed in and around the Santa Maria di Leuca (SML) coral province (Central Mediterranean) has been investigated using different sampling techniques, from dredge and various fishing gears, to a ROV and a lander equipped with video cameras [20]–[22], [13], [10], [23]. D'Onghia et al. [13], sampling benthopelagic fauna in the SML coral province and on muddy bottoms located to the north-west where fishing is fully developed, detected greater abundances and sizes in the former area than in the latter. Thus, these authors detected refuge effects inside the SML coral province and fishing effects outside. Recently, D'Onghia et al. [10] used observations from towed cameras to report that the benthopelagic fauna in the SML coral province is widespread over different meso- and macrohabitats, suggesting the structurally complex habitats represented by coral mounds play a functional role in such a mobile fauna. However, the understanding of the direct role played by the coral habitats on deep-sea fauna distributed in and around the SML coral province is affected by the effect of fishing carried out on this fauna in neighboring sedimentary habitats. In other words, the mobile deep-sea fauna in this geographic area could be less abundant in sedimentary habitats due to fishing effect rather than more abundant in coral habitats due to habitat complexity.

With the aim of detecting the role played by the presence of corals on the deep-sea fish fauna, two longline surveys were carried out in two types of megahabitat within the SML coral province differently characterized by the presence of corals. The term “megahabitat” used in this work refers to a habitat with a range of spatial scale in kilometres which includes different meso-macrohabitats [24], [25]. One type is characterized by complex topography including mesohabitats with carbonate mounds and corals. The other type of megahabitat, although characterized by complex topographic features, lacks carbonate mounds and corals. Both megahabitat typologies are characterized by the virtual absence of fishing or by the same negligible commercial longlining pressure due their complex topography and irregular bottoms not being suitable for trawling [13], [30]. In this work the authors present an analysis of these two surveys with the aim of comparing distribution and abundance of the fish fauna in coral *versus* non-coral megahabitats.

## Materials and Methods

### Study area

**Figure 1:**
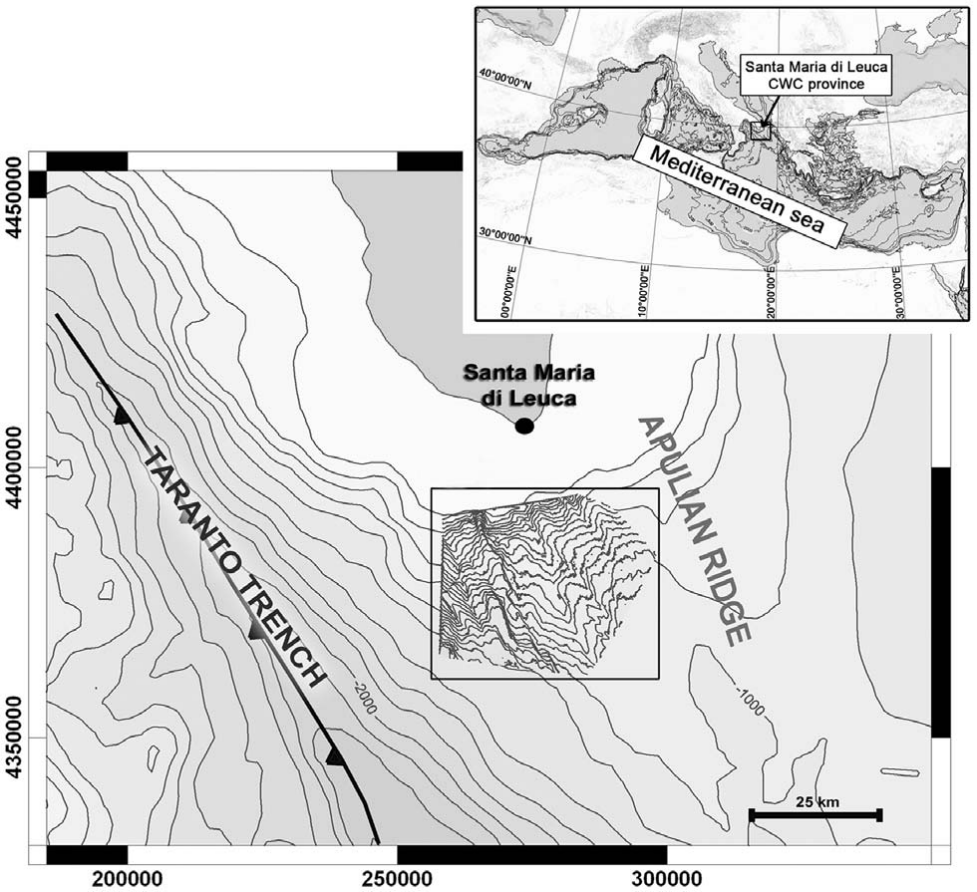
Santa Maria di Leuca cold-water coral (CWC) province in the Mediterranean Sea and bathymetric framework within the southern Apulia margin (APLABES project - Corselli, 2010).

The Santa Maria di Leuca (SML) cold-water coral province is located along the Apulian margin, a few miles off Cape Santa Maria di Leuca (Italy) in the Northern Ionian Sea (Central Mediterranean) (Fig. 1). During the APLABES project [26] an area of 800 km^2^, between approximately 200 and 1300 m in depth, was investigated using a multi-beam echo sounder, side scan sonar, high-resolution seismic profiles and video systems. This area consists of a broad north-eastern sector characterised by mass-transport deposition, with a very complex hummocky seafloor consisting of widespread mound-like reliefs, a central ridge where drift sedimentation was recognised by documenting the action of contour currents from the north-east and a western sector with prominent fault-scarps where widespread erosion processes are evident from the emergence of stiff and/or hardened older sediments [27]. Living colonies of *Madrepora oculata* and *Lophelia pertusa* were collected between 425 and 1100 m in depth [20], [28] and their westernmost presence was recorded by Freiwald et al. [22] using a ROV during the HERMES R/V Meteor M70-1 cruise. Such a presence refers to a vertical escarpment (Gallipoli escarpment) which forms the eastern wall of a major canyon system. Live *Madrepora* and *Lophelia* were recorded at depths between 744-670 m and 744-603 m, respectively [22]. Most probably, the main deep current flowing from the Adriatic Sea into the northern Ionian in a NE-SW direction [29] provides a continuous and regular supply of nutrients and particulate organic carbon to the SML corals which are, indeed, preferentially settled on the top and north-eastern upper flanks of the SML topographic heights.

**Figure 2:**
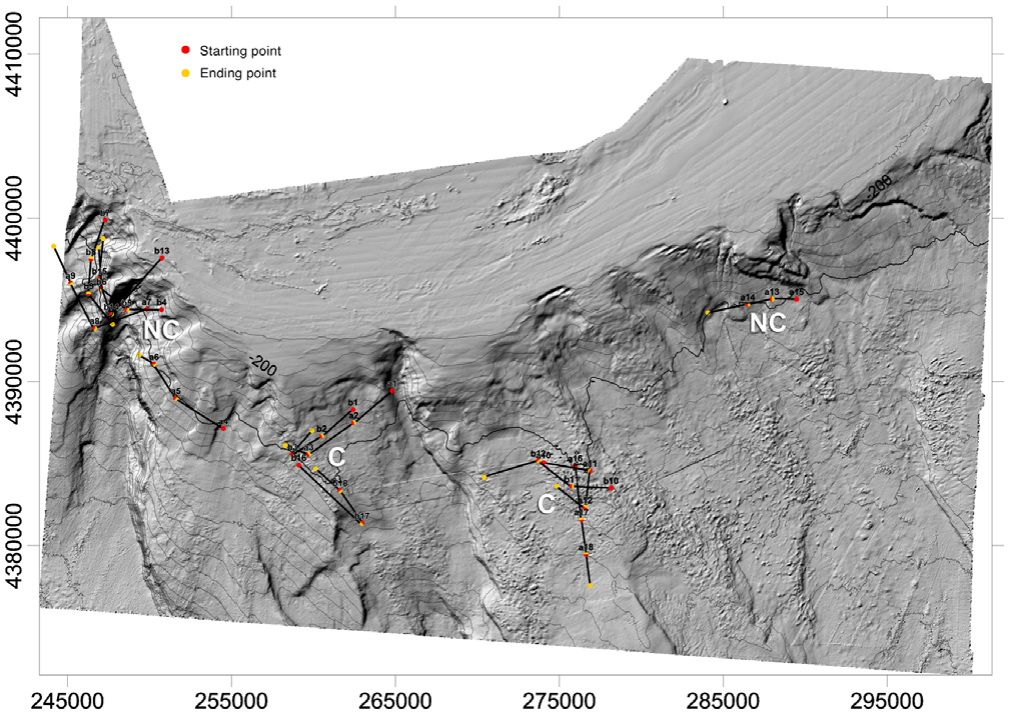
Longline stations in the coral megahabitat (C) and non-coral megahabitat (NC) in the Santa Maria di Leuca cold-water coral province (Northern Ionian Sea) (Map by Savini et al., submitted).

Recently, habitat mapping based on wide area bathymetric and backscatter data recorded as part of the CoralFISH and MAGIC projects, has provided indications of complex topographic features over an area of about 1700 km^2^ between approximately 200 and 1400 m in depth, including the Gallipoli escarpment surveyed by Freiwald et al. [22] (Savini et al. submitted) (Fig. 2).

Considering the marine habitat classification concepts for ecological data management [24], [25], the SML coral province could be considered as a “seascape” which comprises: “megahabitats” at a range of a spatial scale of 1–10 km, including the main seafloor morphologies, such as fault scarpments, troughs and blocky areas [22], [27]; “mesohabitats” at a range of a spatial scale of 10–1000 m, including mud-, coral- and rock-dominated habitats; “macrohabitats” that can be distinguished to a lesser range of spatial scale (1–10 m) [19].

### Survey methodology

Two longline experimental surveys were carried out in the Santa Maria di Leuca (SML) coral province during May–June and September–October 2010. A commercial fishing vessel was hired for the experimental surveys. It had the following characteristics: LFT 14.10 m, GRT 8.97 t; engine power 104.41 kW. The fishing vessel was equipped with a monofilament longline (Table 1). Due to its length, the longline is a fishing gear which allows the capture of benthopelagic fish fauna in a megahabitat. It is a selective gear and its selectivity mostly depend on the size of the hooks. The type of hooks employed were J-hook 7 and J-hook 9. The number 9 J-hook was used with the aim of catching the blackspot seabream *Pagellus bogaraveo* which seems to be a fish species associated with the presence of corals [13], [10].

**Table 1:** Technical characteristics of the bottom longline used in the SML coral province.

Type of gear	monofilament
Length deployed (m)	∼3000
Mean soak time (hour)	4.9±0.3
Bait	fresh *Sardina pilchardus*
Type hook	J-hook
Hook size	7/0 and 9/0
Number of hooks	500/line
Diameter of mainline (mm)	6
Material of mainline	synthetic fiber
Material of snoods	nylon
Snoods distance (m)	5
Length of snoods (m)	2.5
Floats/weights	floats are attached to a big cement weight (about 5 kg) by means of a rope at beginning and end of the main line
Safety line	absent

The sampling was carried out in two types of megahabitat (Fig. 2):

1. a coral megahabitat characterized by a complex topography including mesohabitats with corals (C);
2. a non-coral megahabitat characterized by a complex topography including mesohabitats without corals (NC).

In both megahabitats the depths examined were between 400 and 800 m. Commercial fishing is generally carried out on the northern boundaries of the SML coral province [30]. Thus, both the coral (C) and non-coral megahabitats (NC) considered in this study are only occasionally subject to commercial longlining.

During each survey the sampling was carried out for six days. Each day 3 longlines were employed (2 with number 7 J-hooks and 1 with number 9 J-hooks); the soak time lasted about 5 hours on average and the fishing effort was 1500 hooks/day. A total of 6 hauls with hook size 7 and 3 with hook size 9 were carried out in the two megahabitat typologies during each survey (Table 2 and 3).

**Table 2:** Sampling stations, with mean depth and geographic coordinates in the coral megahabitat (C) and non-coral megahabitat (NC) in the SML coral province during May–June 2010.

			START			END		
Date	Station	Megahabitat	Depth (m)	Latitude (N)	Longitude (E)	Depth (m)	Latitude (N)	Longitude (E)
28/05/2010	a1	C	396	39°37.355	18°15.599	460	39°36.268	18°13.999
	a2	C	460	39°36.268	18°13.999	437	39°35.171	18°12.116
	a3	C	437	39°35.171	18°12.116	499	39°35.271	18°10.570
29/05/2010	a4	NC	487	39°35.948	18°08.457	503	39°36.880	18°06.380
	a5	NC	503	39°36.880	18°06.380	551	39°37.987	18°05.410
	a6	NC	551	39°37.987	18°05.410	512	39°38.270	18°04.761
30/05/2010	a7	NC	561	39°39.790	18°05.029	594	39°39.103	18°02.848
	a8	NC	594	39°39.103	18°02.848	561	39°40.571	18°01.750
	a9	NC	561	39°40.571	18°01.750	503	39°41.301	18°00.721
31/05/2010	a10	C	512	39°35.135	18°22.134	524	39°34.916	18°24.122
	a11	C	524	39°34.916	18°24.122	594	39°33.692	18°23.950
	a12	C	594	39°33.692	18°23.950	545	39°34.375	18°22.726
07/06/2010	a13	NC	450	39°40.740	18°31.660	470	39°40.230	18°28.950
	a14	NC	470	39°40.230	18°28.950	450	39°39.260	18°28.360
	a15	NC	450	39°39.260	18°28.360	460	39°38.430	18°27.290
08/06/2010	a16	C	550	39°35.060	18°23.480	580	39°33.290	18°23.810
	a17	C	580	39°33.290	18°23.810	620	39°32.150	18°24.050
	a18	C	620	39°32.150	18°24.050	650	39°31.130	18°24.270

**Table 3:** Sampling stations, with mean depth and geographic coordinates in the coral megahabitat (C) and non-coral megahabitat (NC) in the SML coral province during September–October 2010.

			START			END		
Date	Station	Megahabitat	Depth (m)	Latitude (N)	Longitude (E)	Depth (m)	Latitude (N)	Longitude (E)
1710/2010	b1	C	404	39°37.355	18°15.599	431	39°36.268	18°13.999
	b2	C	431	39°36.268	18°13.999	479	39°35.171	18°12.116
	b3	C	479	39°35.171	18°12.116	470	39°35.271	18°10.570
29/09/2010	b4	NC	430	39°35.948	18°08.457	594	39°36.880	18°06.380
	b5	NC	594	39°36.880	18°06.380	462	39°37.987	18°05.410
	b6	NC	462	39°37.987	18°05.410	495	39°38.270	18°04.761
23/09/2010	b7	NC	414	39°39.790	18°05.029	512	39°39.103	18°02.848
	b8	NC	512	39°39.103	18°02.848	552	39°40.571	18°01.750
	b9	NC	552	39°40.571	18°01.750	577	39°41.301	18°00.721
15/10/2010	b10	C	528	39°35.135	18°22.134	533	39°34.916	18°24.122
	b11	C	533	39°34.916	18°24.122	524	39°33.692	18°23.950
	b12	C	524	39°33.692	18°23.950	552	39°34.375	18°22.726
24/09/2010	b13	NC	363	39°40.740	18°31.660	594	39°40.230	18°28.950
	b14	NC	594	39°40.230	18°28.950	495	39°39.260	18°28.360
	b15	NC	495	39°39.260	18°28.360	487	39°38.430	18°27.290
16/10/2010	b16	C	528	39°35.060	18°23.480	668	39°33.290	18°23.810
	b17	C	668	39°33.290	18°23.810	530	39°32.150	18°24.050
	b18	C	530	39°32.150	18°24.050	467	39°31.130	18°24.270

### Data analysis

Total length (TL) (mm), weight (g) and sex were recorded for each specimen collected. Results on sex are not reported in the present study. According to Durán Muñoz et al. [31], the catch per unit effort (CPUE) in number (N) and biomass (kg) was calculated as a relative index of abundance, following the equation: CPUE = catch in N/1000 hooks and kg/1000 hooks on the longline. With the aim of estimating the variability of the CPUE between the sampling stations, the average CPUE value for each survey was calculated as follows: ∑ CPUE_i_/n; where CPUE_i_ is the catch per unit effort of each station and n is the number of stations in each survey.

Coral by-catch of colonial cold-water coral species was recorded for each sampling station. Both entire colonies and pieces or fragments of colonies were counted by species and identified as living or dead (dark-coated) corals. Moreover, following the paper by Sampaio et al. [32], the total length of the entire colonies was measured and 3 size classes were identified as small colony (length <20 cm), medium colony (20 cm<length<50 cm) and large colony (>50 cm). The frequency of coral occurrence (F%) in each station was computed as the percentage of hooks with corals of the total number of hooks employed.

The differences in the total catch and in the capture of the most abundant fish species, both in number and biomass, between coral and non-coral megahabitats, were evaluated using the Mann Whitney U-test [33]. Since most fish species caught in the SML coral province do not migrate seasonally [34]–[38], no statistical tests were carried out to evaluate any differences between the two seasons.

Multivariate analysis was performed in order to detect significant differences between the faunal assemblage in coral (C) and non-coral megahabitats (NC). Matrices of relative abundance index per species-station (CPUE), both in number (N/1000 hooks) and biomass (kg/1000 hooks), were compiled using original data and fourth root transformation. Ordination of the sampling stations was performed by means of non metric MultiDimensional Scaling (nMDS), based on the Bray-Curtis similarity index using PRIMER 6 software [39]. The nMDS preserves the rank order of the inter-sample distance, as opposed to the linear relationship of classical metric scaling. This analysis is not sensitive to outliers and has been widely used to explain the space ordination of samples [39]. Moreover, the stress values obtained from nMDS have been utilized as an adequacy measure of representation for two-dimensional ordination (preservation of the original inter-sample distance, increasing adequacy-decreasing stress value) in order to minimise mis-interpretation of data [39]. ANalysis Of SIMilarities (ANOSIM) was applied to test the differences between the station-groups identified by the nMDS analysis. SIMPER (similarity percentages) was employed to identify the species that contributed most to the observed dissimilarities between groups in relation to megahabitat type.

The number of individuals and size-range of each species captured were recorded. Length-frequency distributions were computed for the most abundant fish species and the differences between coral and non-coral megahabitat distributions were evaluated using the Kolmogoroff-Smirnov test [33].

## Results

### Species abundance and diversity pattern

A total of 17 fish species (13 C and 12 NC) were identified out of a total of 781 specimens (400 C and 381 NC) and 19 fish species (18 C and 10 NC) from a total of 659 specimens (357 C and 302 NC) collected during the first and second longline surveys respectively. Considering the species with a demersal habit, the cartilagineous fish *Leucoraja fullonica* and *Pteroplatytrygon violacea* represent new records for the SML coral province.

The average CPUE values, in number and biomass, of each species and the whole sample are presented in Tables 4 and 5. No significant differences were detected in the average total catch per unit effort, either in number or biomass, between the two sampled megahabitats. In both surveys, the most abundant cartilagineous fish was the blackmouth catshark *Galeus melastomus*. Its abundance was greater in the non-coral than in the coral megahabitat although a high variability in the catch was observed and no significant differences were detected. The most abundant teleosts in both megahabitats were *Conger conger*, *Helicolenus dactylopterus*, *Merluccius merluccius*, *Pagellus bogaraveo*, *Phycis blennoides* and *Polyprion americanus*. High variability in the catch was also observed for these fishes. The skates *Dipturus oxyrinchus* and *Leucoraja fullonica* and the blackspot seabrem *P. bogaraveo* were only found in the coral megahabitat. During both surveys, the CPUE values in number of *H. dactylopterus* and *C. conger* were significantly greater in the coral than in the non-coral megahabitat. During the second survey, *P. americanus* was caught with significantly greater CPUE values, both in number and biomass, in the coral megahabitat.

**Table 4:** Average CPUE (N/1000 hooks and kg/1000 hooks) per species and average total CPUE obtained in the coral megahabitat (C) and non-coral megahabitat (NC) in the SML coral province during May–June 2010 (s.d. = standard deviation; * = p<0.05).

	N/1000 hooks				kg/1000 hooks			
	C		NC		C		NC	
	Mean	± s.d.	Mean	± s.d.	Mean	± s.d.	Mean	± s.d.
**Chondrichthyes**								
*Centrophorus granulosus* (Gulper shark)	-	-	0.22	0.67	-	-	0.47	1.41
*Dipturus oxyrinchus* (Longnose skate)	0.22	0.67	-	-	1.44	4.33	-	-
*Etmopterus spinax* (Velvet belly)	1.56	2.60	2.00	3.61	0.28	0.51	0.34	0.65
*Galeus melastomus* (Blackmouth catshark)	26.67	31.53	48.00	62.74	8.55	10.26	13.96	18.46
*Leucoraja circularis* (Sandy ray)	-	-	0.22	0.67	-	-	0.56	1.67
*Pteroplatytrygon violacea* (Pelagic stingray)	-	-	0.22	0.67	-	-	0.44	1.33
**Osteichthyes**								
*Brama brama* (Atlantic pomfret)	-	-	0.44	0.88	-	-	0.76	1.53
*Conger conger* (European conger)	12.44 *	8.17	8.22	15.08	16.05	14.82	36.01	70.15
*Helicolenus dactylopterus* (Blackbelly rosefish)	22.44 *	18.02	7.56	9.68	4.36	3.43	2.01	2.53
*Lepidopus caudatus* (Silver scabbardfish)	0.22	0.67	-	-	0.27	0.80	-	-
*Merluccius merluccius* (European hake)	8.00	4.69	5.78	4.52	15.05	11.96	7.55	5.27
*Micromesistius poutassou* (Blue whiting)	0.89	1.05	2.22	2.11	0.29	0.37	0.62	0.61
*Molva dipterygia* (Blue ling)	0.44	0.88	-	-	0.31	0.65	-	-
*Mora moro* (Common mora)	0.44	1.33	-	-	0.34	1.01	-	-
*Pagellus bogaraveo* (Blackspot seabream)	6.67	9.75	-	-	2.65	3.77	-	-
*Phycis blennoides* (Greater forkbeard)	8.22	8.03	8.22	9.82	3.72	4.04	5.58	6.36
*Polyprion americanus* (Wreckfish)	0.67	1.00	1.78	3.93	2.98	4.73	9.45	24.19
**Average Total CPUE**	**88.89**	**60.13**	**84.89**	**69.13**	**56.28**	**32.20**	**73.76**	**96.85**

**Table 5:** Average CPUE (N/1000 hooks and kg/1000 hooks) per species and average total CPUE obtained in the coral megahabitat (C) and non-coral megahabitat (NC) in the SML coral province during September–October 2010 (s.d. = standard deviation; * = p<0.05).

	N/1000 hooks				kg/1000 hooks			
	C		NC		C		NC	
	Mean	± s.d.	Mean	± s.d.	Mean	± s.d.	Mean	± s.d.
**Chondrichthyes**								
*Centrophorus granulosus* (Gulper shark)	0.44	0.88	0.44	1.33	1.40	2.78	1.28	3.84
*Dipturus oxyrinchus* (Longnose skate)	0.44	0.88	-	-	2.23	5.63	-	-
*Etmopterus spinax* (Velvet belly)	3.11	3.02	3.56	6.31	0.31	0.33	0.65	1.18
*Galeus melastomus* (Blackmouth catshark)	19.11	13.86	30.00	31.30	6.01	3.96	8.41	8.25
*Leucoraja circularis* (Sandy ray)	0.44	1.33	-	-	1.12	3.37	-	-
*Leucoraja fullonica* (Shagreen ray)	0.89	1.05	-	-	1.65	3.24	-	-
*Prionace glauca* (Blue shark)	0.22	0.67	-	-	1.33	4.00	-	-
*Pteroplatytrygon violacea* (Pelagic stingray)	1.33	1.73	-	-	1.93	2.46	-	-
**Osteichthyes**								
*Brama brama* (Atlantic pomfret)	0.44	1.33	-	-	0.78	2.33	-	-
*Conger conger* (European conger)	10.00 *	8.54	2.89	4.14	15.25	16.25	13.56	37.25
*Helicolenus dactylopterus* (Blackbelly rosefish)	23.11 *	20.33	10.44	10.14	5.60	4.84	2.56	2.44
*Lepidopus caudatus* (Silver scabbardfish)	-	-	1.33	3.32	-	-	2.02	4.75
*Merluccius merluccius* (European hake)	3.56	2.96	6.00	2.45	4.60	4.00	8.14	4.56
*Micromesistius poutassou* (Blue whiting)	3.33	4.12	2.67	3.16	1.01	1.29	0.84	1.00
*Molva dipterygia* (Blue ling)	0.22	0.67	-	-	0.29	0.87	-	-
*Pagellus bogaraveo* (Blackspot seabream)	5.11	8.72	-	-	2.25	3.83	-	-
*Phycis blennoides* (Greater forkbeard)	5.56	3.57	9.56	6.31	2.71	1.58	5.30	3.82
*Polyprion americanus* (Wreckfish)	1.78 *	2.11	0.22	0.67	5.03 *	5.64	0.49	1.48
*Xiphias gladius* (Swordfish)	0.22	0.67	-	-	0.67	2.00	-	-
**Average Total CPUE**	**79.33**	**39.47**	**67.11**	**47.99**	**54.16**	**24.91**	**43.30**	**50.68**

**Figure 3:**
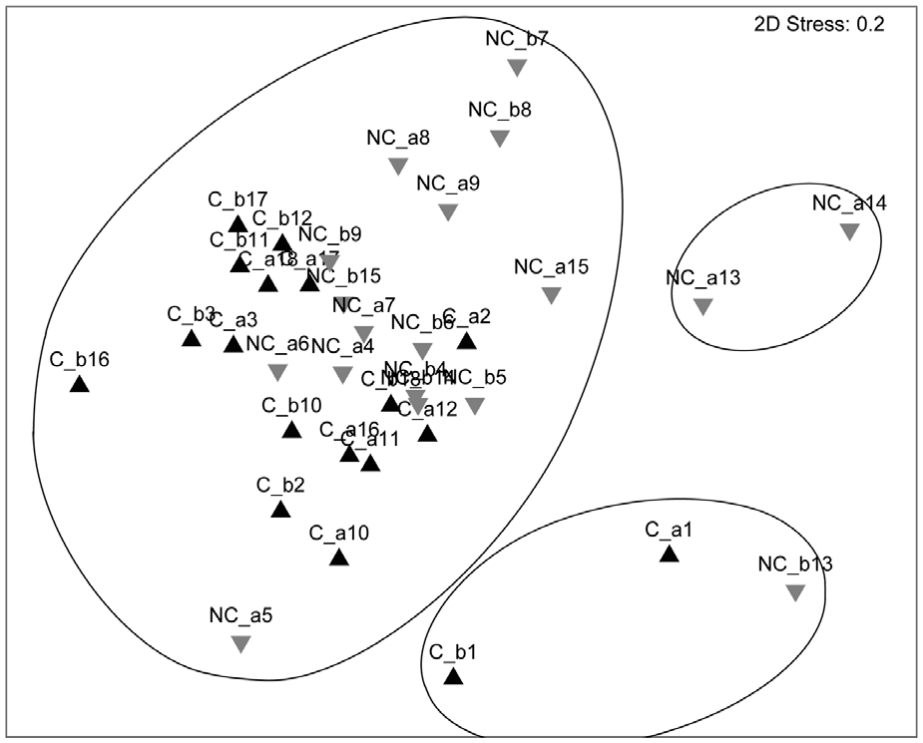
Non-parametric multidimensional scaling of relative abundance index in number (N/1000 hooks) computed for all the stations carried out in the SML coral province (▴ = coral megahabitat; ▾ = non-coral megahabitat) (Fourth root transformation; Global R = 0.14, not significant).

**Figure 4:**
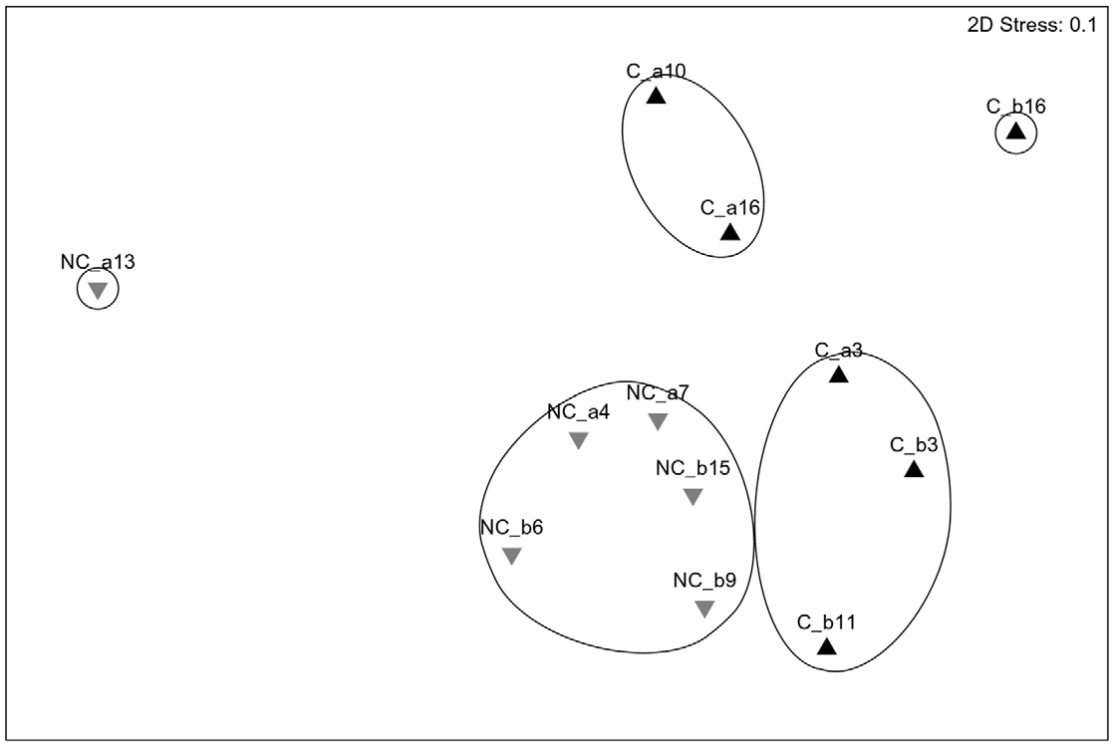
Non-parametric multidimensional scaling of relative abundance index in number (N/1000 hooks) computed for the stations using J-hook 9 in the SML coral province (▴ = coral megahabitat; ▾ = non-coral megahabitat) (Fourth root transformation; Global R = 0.33; p<0.005).

With regard to the multivariate analysis, considering all the sampling stations and the two types of hook, no significant differences between the fish assemblage sampled in coral and non-coral megahabitats were observed both using transformed and original data (Fig. 3, Table 6). Considering the sampling stations related to the longlines with number 9 J-hooks, significant differences between the fish assemblage sampled in coral and non-coral megahabitats were detected in abundance index, for both number and biomass using transformed data and only in abundance index for number using rough data (Fig. 4, Table 6). The stress value of 0.1 indicates that sample points fit well in the low-dimensional ordination space. The stations in the coral megahabitats were characterized by the presence of *P. bogaraveo* which was not found in the non-coral megahabitat stations. In fact, *P. bogaraveo* contributed most to significant dissimilarity in assemblages between coral and non-coral megahabitats both in terms of density and biomass. The other species mainly contributing to the dissimilarity between the two group-stations were *G. melastomus*, *E. spinax*, *H. dactylopterus* for density and *C. conger*, *P. americanus*, *G. melastomus* for biomass (Table 7).

**Table 6:** Results of the ANOSIM global test carried out for CPUE values (relative abundance indices) obtained in the coral and non-coral megahabitat in the SML coral province (ns = not significant).

	Transformed data		No transformed data	
	CPUE	CPUE	CPUE	CPUE
	(N/1000 hooks)	(kg/1000 hooks)	(N/1000 hooks)	(kg/1000 hooks)
**All stations**	R = 0.14 ns	R = 0.13 ns	R = 0.11 ns	R = 0.09 ns
**Hook 9 stations**	R = 0.33 p<0.005	R = 0.25 p<0.05	R = 0.31 p<0.05	R = 0.11 ns

**Table 7:** SIMPER analysis of density (N/1000 hooks) and biomass (kg/1000 hooks) computed for the stations using J-hook 9 in the SML coral province.

Density average dissimilarity = 37.36						
	Group IN	Group OUT				
Species	Av. Abund	Av. Abund	Av. Diss	Diss/SD	Contrib%	Cum.%
*Pagellus bogaraveo*	1.69	0.00	7.14	1.90	19.11	19.11
*Galeus melastomus*	1.92	2.23	4.42	1.36	11.84	30.95
*Etmopterus spinax*	0.98	0.84	3.64	1.20	9.75	40.70
*Helicolenus dactylopterus*	2.24	1.67	3.43	0.86	9.19	49.90
*Micromesistius poutassou*	0.97	1.14	2.90	1.07	7.77	57.66
*Conger conger*	1.94	1.30	2.71	1.01	7.26	64.92
*Phycis blennoides*	1.35	1.65	2.50	0.90	6.68	71.61
*Polyprion americanus*	0.66	0.20	2.40	0.99	6.42	78.02
*Merluccius merluccius*	1.15	1.43	1.74	0.78	4.65	82.67
*Brama brama*	0.24	0.20	1.53	0.60	4.11	86.78
*Pteroplatytrygon violacea*	0.43	0.00	1.53	0.68	4.10	90.88

Biomass average dissimilarity = 37.83						
	Group IN	Group OUT				
Species	Av. Abund	Av. Abund	Av. Diss	Diss/SD	Contrib%	Cum.%
*Pagellus bogaraveo*	1.31	0.00	6.40	1.91	16.91	16.91
*Conger conger*	1.97	1.84	3.75	1.11	9.91	26.81
*Polyprion americanus*	0.89	0.25	3.69	1.02	9.77	36.58
*Galeus melastomus*	1.49	1.62	3.35	1.24	8.87	45.44
*Etmopterus spinax*	0.54	0.55	2.58	1.27	6.82	52.26
*Micromesistius poutassou*	0.74	0.84	2.55	1.12	6.75	59.02
*Helicolenus dactylopterus*	1.44	1.18	2.55	0.95	6.73	65.75
*Phycis blennoides*	1.08	1.49	2.51	0.82	6.63	72.37
*Merluccius merluccius*	1.27	1.50	2.37	0.90	6.27	78.65
*Brama brama*	0.27	0.22	1.99	0.60	5.27	83.92
*Centrophorus granulosus*	0.26	0.31	1.91	0.62	5.04	88.96
*Pteroplatytrygon violacea*	0.47	0.00	1.87	0.68	4.95	93.91

**Figure 5:**
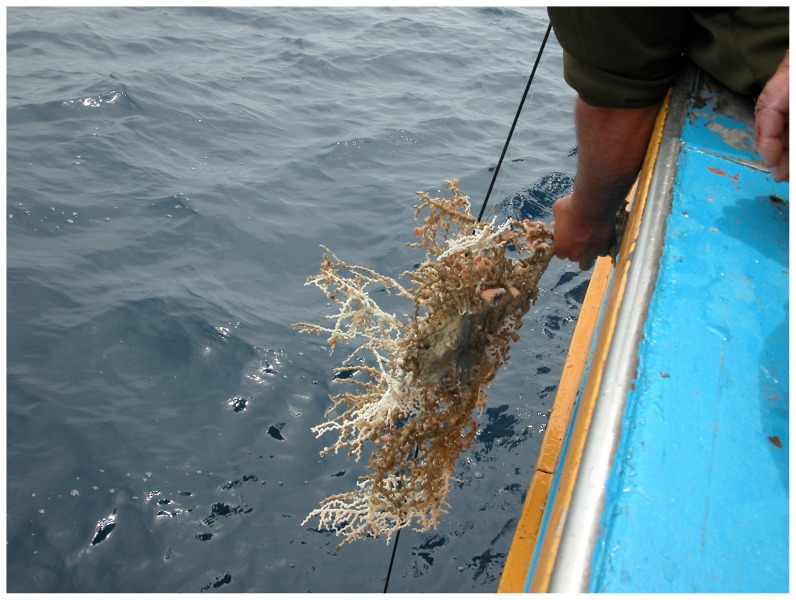
A live colony of *Madrepora oculata* with the presence of the solitary *Desmophillum dianthus* corals collected by longline in the coral megahabitat of the SML cold-water coral province.

**Figure 6:**
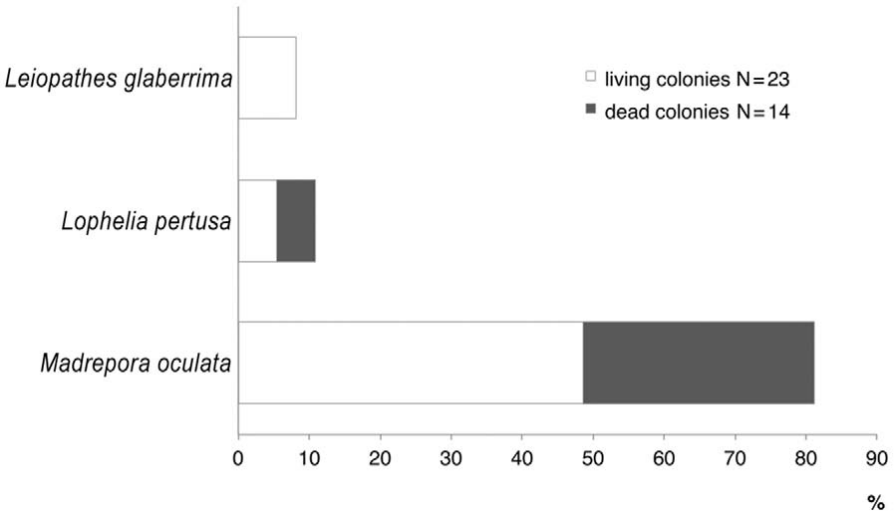
Percentage of colonies of cold-water coral species collected by longline in the coral megahabitat of the SML coral province.

**Figure 7:**
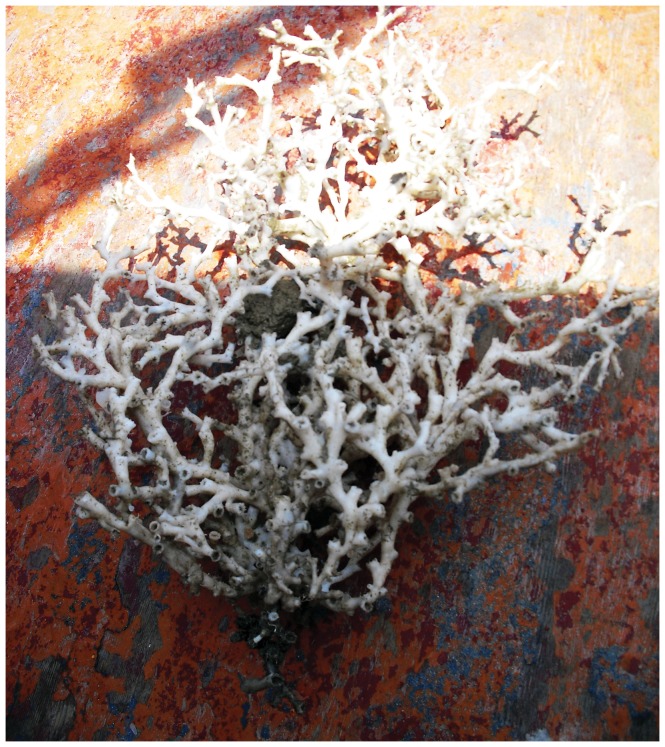
Colony of *Lophelia pertusa* collected by longline in the coral megahabitat of the SML cold-water coral province.

**Figure 8:**
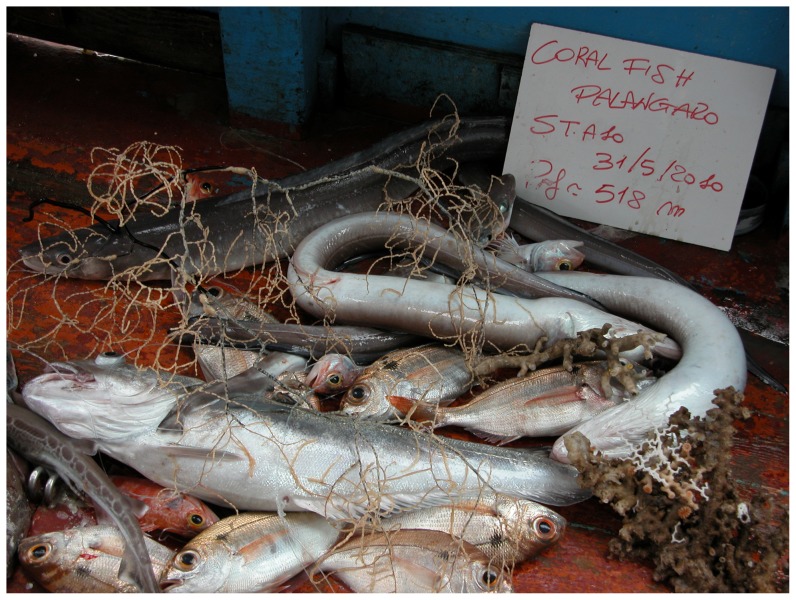
Colonies of *Madrepora oculata* and *Leiopathes glaberrima* collected by longline in the coral megahabitat of the SML cold-water coral province.

The coral by-catch was only obtained in the coral megahabitat in about 55% of the stations investigated in this type of megahabitat in both surveys (Table 8). All specimens were directly entangled in the longline (Fig. 5). A total of 37 colonies belonging to 3 species (*Leiopathes glaberrima*, *Lophelia pertusa* and *Madrepora oculata*) were accidentally collected: 23 of them were living and the other 14 appeared dark-coated and dead (Fig. 6). *M. oculata* was the most abundant cold-water coral in the by-catch, with a maximum number of 7 colonies per station. Most of the colonies were medium in size; however, all three size-classes were present in the catch. *L. pertusa* (Fig. 7) and the black coral *L. glaberrima* (Fig. 8) were collected in few stations with only one complete colony and/or one piece. All the entire colonies were branched and had a 3-dimensional structure. The frequency of occurrence (F%) ranged from 0.4 to 2.2 (Table 8). In stations a3, b10 and b16 two hooks were entangled on the same colony. On the colonial scleractinian species, both live and dead, the presence of *Desmophillum dianthus* was frequently recorded but data on number and size are not reported in this paper.

**Table 8:** Number of colonies (N) and pieces (n) by size class and frequency of occurrence (F%) of coral by-catch for each station in coral megahabitat in the SML coral province during May–June and September–October 2010.

Survey	Station	*Leiopathes glaberrima*				*Lophelia pertusa*				*Madrepora oculata*				Total		
			Colonies		pieces		Colonies		pieces		Colonies		pieces	N	n	F%
		small	medium	large		small	medium	large		small	medium	large		colonies	pieces	
May–June	a3	-	-	-	-	-	-	-	-	-	1	-	-	**1**	**0**	**0.4**
2010	a10	-	-	1	-	1	-	-	-	-	1	-	-	**3**	**0**	**0.6**
	a11	-	-	-	-	-	1	-	1	-	5	2	2	**8**	**3**	**2.2**
	a16	-	-	-	-	-	1	-	-	-	4	1	-	**6**	**0**	**1.2**
	a17	-	-	-	-	-	-	1	-	-	3	-	6	**4**	**6**	**2.0**
September–October	b2	-	-	-	-	-	-	-	-	1	-	-	1	**1**	**1**	**0.4**
2010	b10	-	-	-	-	-	-	-	-	2	2	-	2	**4**	**2**	**1.4**
	b12	-	-	-	-	-	-	-	-	-	2	-	-	**2**	**0**	**0.4**
	b16	-	1	-	1	-	-	-	-	1	-	1	3	**3**	**4**	**1.8**
	b18	-	1	-	1	-	-	-	-	4	-	-	2	**5**	**3**	**1.6**
	**Total**	**-**	**2**	**1**	**2**	**1**	**2**	**1**	**1**	**8**	**18**	**4**	**16**	**37**	**19**	**1.2**

### Size distributions

**Figure 9:**
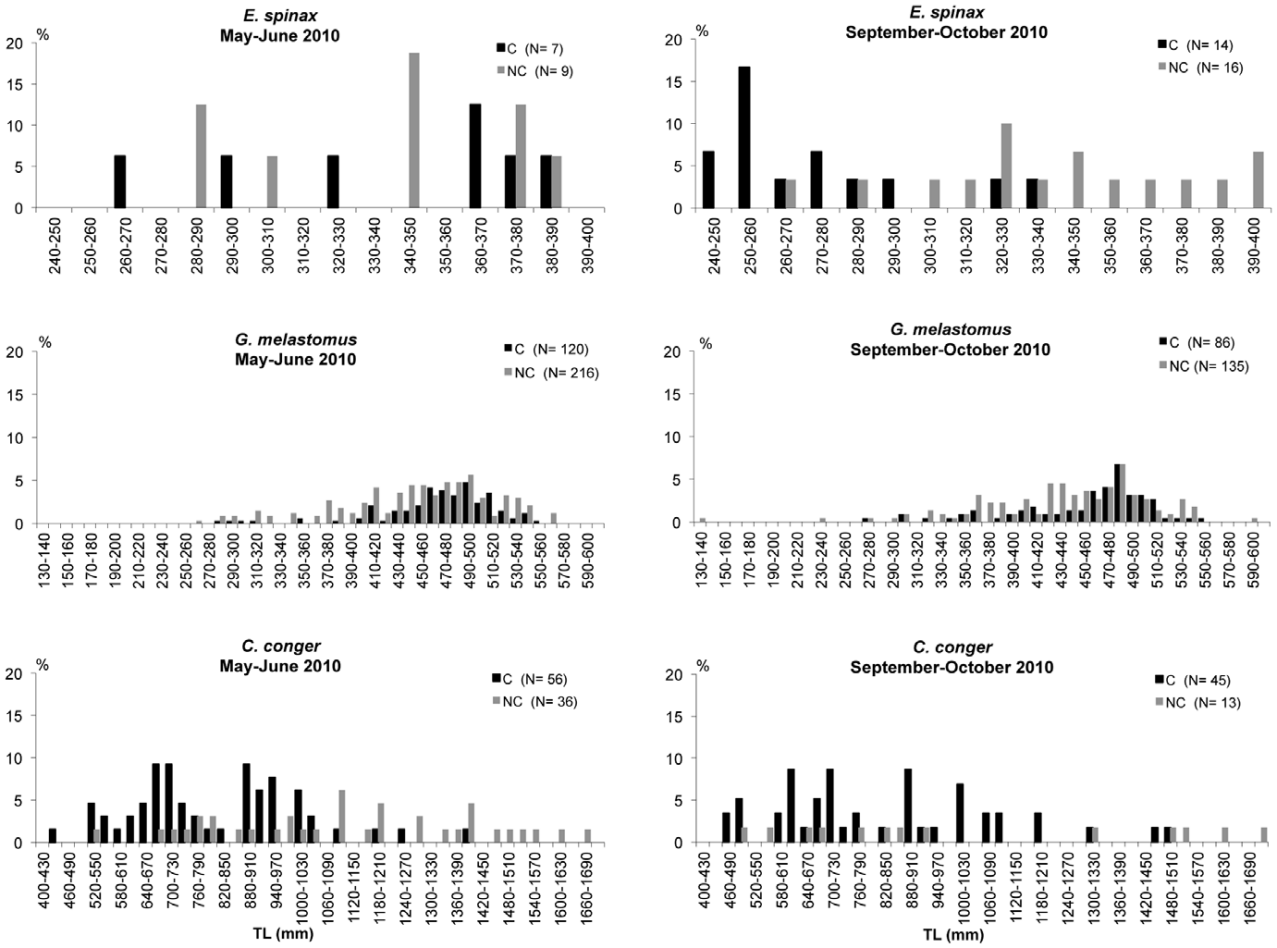
Length-frequency distribution of *Etmopterus spinax*, *Galeus melastomus* and *Conger conger* in the coral megahabitat (C) and non-coral megahabitat (NC) in the SML cold-water coral province.

**Figure 10:**
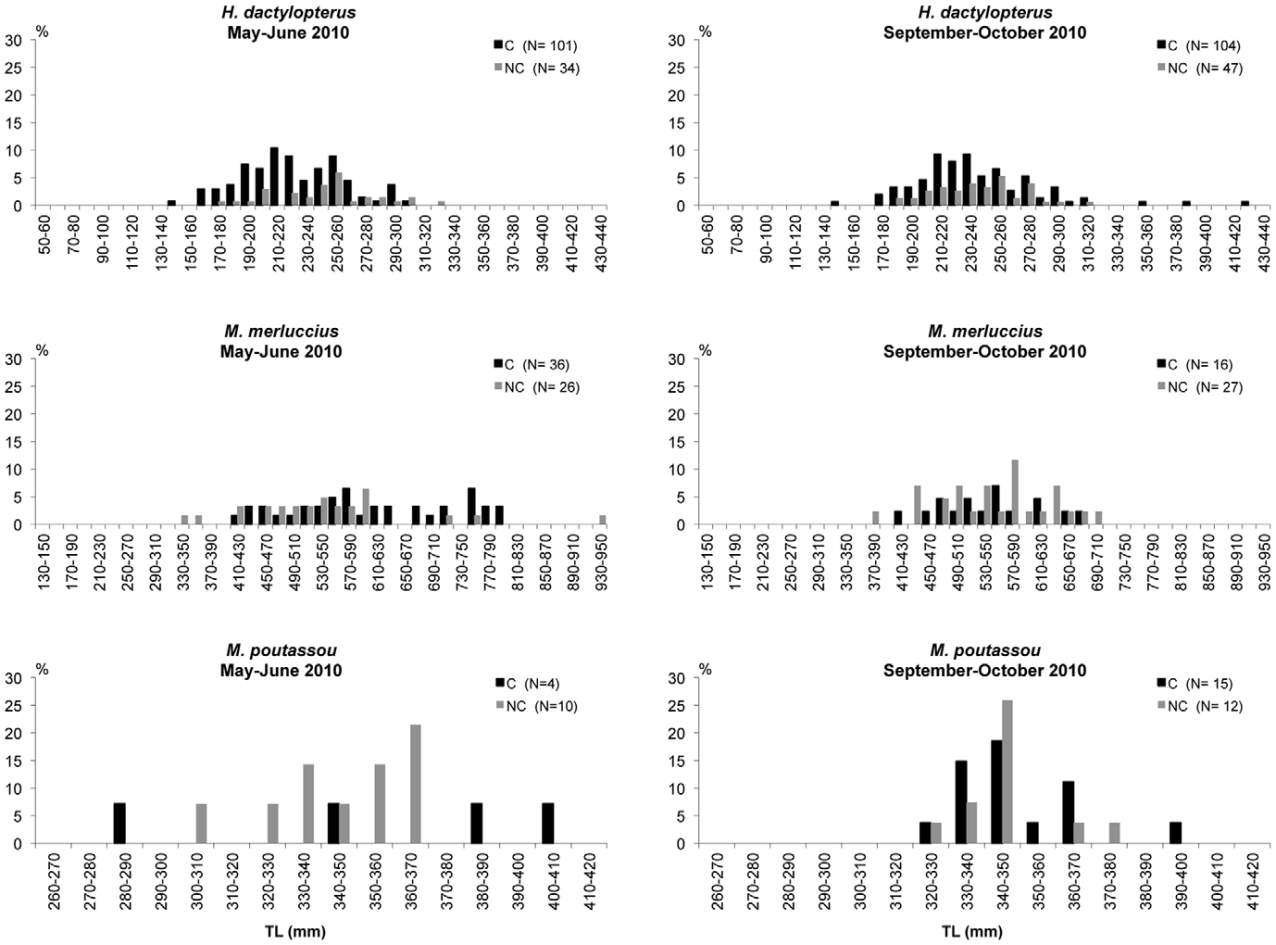
Length-frequency distribution of *Helicolenus dactylopterus*, *Merluccius merluccius* and *Micromesistius poutassou* in the coral megahabitat (C) and non-coral megahabitat (NC) in the SML cold-water coral province.

**Figure 11:**
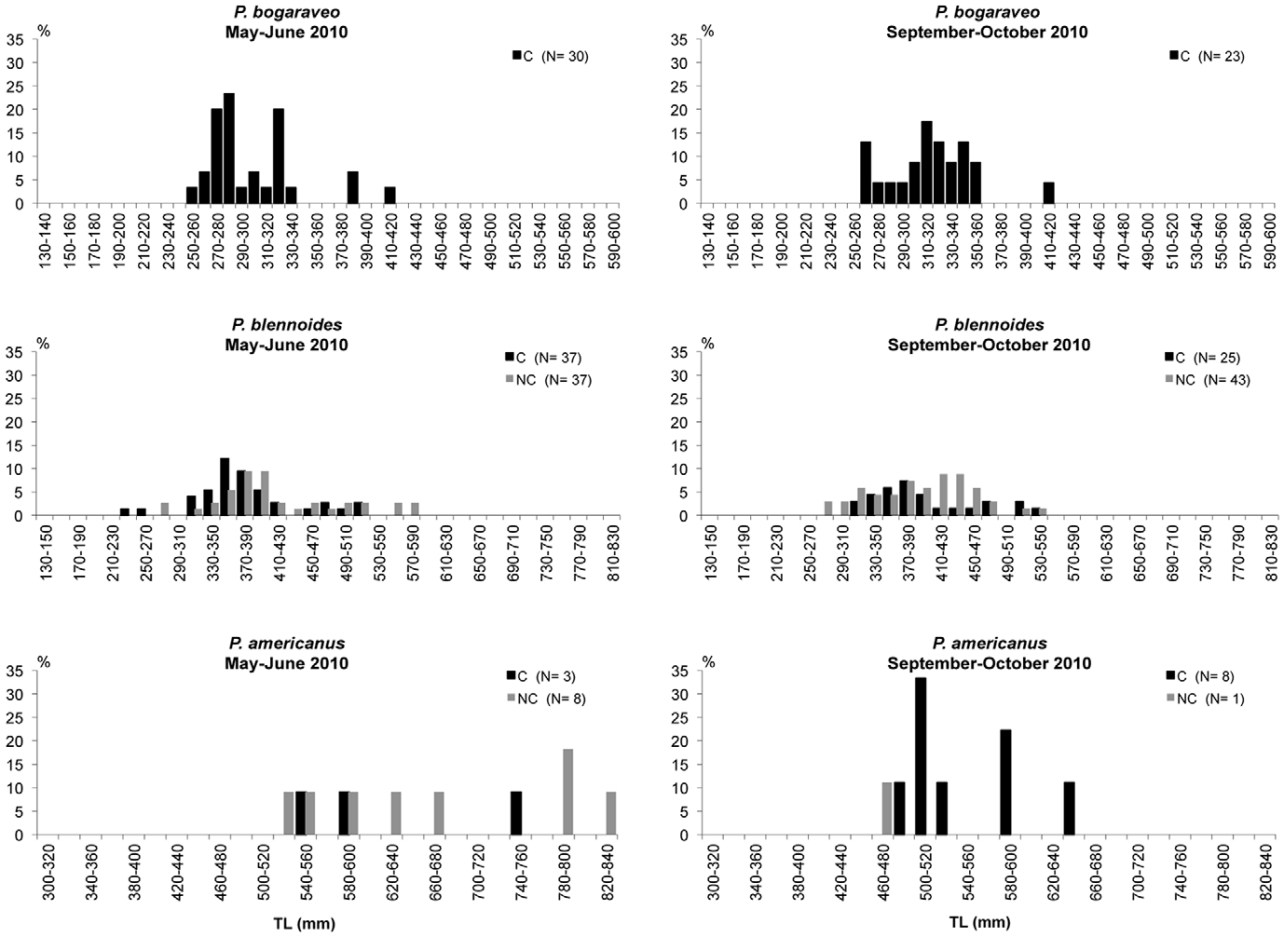
Length-frequency distribution of *Pagellus bogaraveo*, *Phycis blennoides* and *Polyprion americanus* in the coral megahabitat (C) and non-coral megahabitat (NC) in the SML cold-water coral province.

The number of individuals and size-range of each species captured are presented in Table 9. The size distributions of the most abundant fish species are presented in Fig. 9, 10 and 11. The results of the Kolmogoroff-Smirnov test are reported in Table 10.

**Table 9:** Number of individuals (N) and size-range of fish species captured in the SML coral province (C = coral megahabitat; NC = non-coral megahabitat).

	May–June 2010				September–October 2010			
	C		NC		C		NC	
	N	size-range TL (mm)	N	size-range TL (mm)	N	size-range TL (mm)	N	size-range TL (mm)
**Chondrichthyes**								
*Centrophorus granulosus*			1	674	2	780–794	2	773–823
*Dipturus oxyrinchus*	1	1005			2	695–1400		
*Etmopterus spinax*	7	265–387	9	283–380	14	240–334	16	260–398
*Galeus melastomus*	120	284–550	216	265–562	86	275–554	135	130–590
*Leucoraja circularis*			1	812	2	770–800		
*Leucoraja fullonica*					4	490–920		
*Prionace glauca*					1	1200		
*Pteroplatytrygon violacea*			1	940	6	939–1083		
**Osteichthyes**								
*Brama brama*			2	478–600	2	444–590		
*Conger conger*	56	450–1400	36	527–1670	45	475–1500	13	494–1700
*Helicolenus dactylopterus*	101	140–306	34	176–325	104	143–424	47	180–315
*Lepidopus caudatus*	1	1010					6	1035–1270
*Merluccius merluccius*	36	418–800	26	346–933	16	416–686	27	383–690
*Micromesistius poutassou*	4	285–403	10	300–361	15	324–392	12	324–370
*Molva dipterygia*	2	660–730			1	730		
*Mora moro*	2	415–437						
*Pagellus bogaraveo*	30	242–403			23	250–400		
*Phycis blennoides*	37	230–528	37	274–578	25	316–540	43	280–533
*Polyprion americanus*	3	546–740	8	530–824	8	490–647	1	477
*Xiphias gladius*					1	1000		

**Table 10:** Results of the Kolmogoroff-Smirnov test applied to the most abundant fish species collected in the SML coral province (D = statistic value; p = significance: *** p<0.001,** p<0.01, * p<0.05; ns: not significant).

	May–June 2010		September–October 2010	
Species	D	p	D	p
*Etmopterus spinax*	0.238	ns	0.732	***
*Galeus melastomus*	0.224	***	0.215	*
*Conger conger*	0.516	***	0.318	ns
*Helicolenus dactylopterus*	0.309	*	0.100	ns
*Merluccius merluccius*	0.187	ns	0.144	ns
*Micromesistius poutassou*	0.500	ns	0.217	ns
*Phycis blennoides*	0.270	ns	0.175	ns
*Polyprion americanus*	0.375	ns	1.000	ns

The velvet belly *E. spinax* was caught with comparable numbers in the two types of megahabitat. Highly significant differences in the sizes between the two megahabitats were only detected in the second survey when a greater number of medium-small individuals was captured in the coral megahabitat and a greater number of medium-large individuals in the non-coral megahabitat. The blackmouth catshark *G. melastomus* was captured in both megahabitats with a multi-modal size distribution mostly made up of individuals with sizes between 440 and 540 mm in total length. A greater fraction of large and medium-small individuals were collected in the non-coral megahabitat. Significant differences between the two megahabitats were detected in both surveys. *C. conger* was mainly collected in the coral megahabitat with individuals generally smaller than 1000 mm TL. During the first survey highly significant differences between the two megahabitats were detected. The rockfish *H. dactylopterus* was sampled with a wider size-range in the coral than in the non-coral megahabitat during both surveys; however, significant differences between the two megahabitats were only detected during May-June 2010. *M. merluccius*, *Micromesistius poutassou* and *P. blennoides* were caught with comparable size distributions in the two megahabitats. No significant differences emerged from the Kolmogoroff-Smirnov test for any of these three species. The blackspot seabrem *P. bogaraveo* was only captured inside the coral habitat with sizes between 250 and 420 mm TL during May–June and between 260 and 420 mm TL during September–October. *P. americanus* did not show significant differences in the sizes between the two megahabitats.

## Discussion

The fish fauna examined in this study was collected using longline on bottoms with a complex topography, characterized by the presence or absence of corals. Mastrototaro et al. [28], as part of six cruises when 10 different types of sampling gear were used, reported a list of 202 species within 222 taxa identified. D'Onghia et al. [10], using towed cameras, added 8 new species records for the SML coral province and 4 depth records for the Ionian Sea. The present study provides further new records for the SML coral province: the cartilagineous fish *L. fullonica* and *P. violacea*. This updates the biodiversity of the SML coral province confirming that the knowledge on the species diversity of a certain ecosystem is closely related to the number of surveys conducted and types of sampling gear used.

The total catches and the abundance indices in several species, both in number and biomass, were comparable between the two habitat typologies. The species which revealed significant differences between the two investigated megahabitats were the blackspot seabream *P. bogaraveo*, exclusively collected in the coral megahabitat, the conger eel *C. conger*, the rockfish *H. dactylopterus* and the wreckfish *P. americanus* that were found with greater abundance in the coral than the non-coral megahabitats. Differences in size between the two megahabitats were detected in *E. spinax*, *G. melastomus*, *C. conger* and *H. dactylopterus*.

The presence of large specimens of *P. bogaraveo* seems to be exclusive to the coral megahabitat. The fishermen who work off Cape Santa Maria di Leuca state that the coral area is an attractive fishing ground for large individuals of this fish [13] whereas small individuals are usually caught by trawling on muddy bottoms of the northern Ionian Sea [40], [41]. Using towed cameras *P. bogaraveo* was observed in different macrohabitats of the SML coral province; however, the distribution of adult specimens seems to be associated with the presence of corals [10, present work]. This fish feeds both near the bottom on benthic prey and in the water column on pelagic species [42], [43]. The corals, as living structures protruding from the seafloor, increase the habitat complexity, modifying the hydrodynamics, providing firm substrata both for larval settlement and adult organisms, increasing food sources and contributing to the species richness [4], [9]. In the SML coral province the megahabitats with corals are also rich in sponges [28] which themselves create a complex living space for a large number of species from many taxa [44]. Although both corals and sponges have facultative symbionts [45], [44], several species belonging to copepods, amphipods, isopods and decapods consume these symbionts [9]. As a result, such a high species richness enhances the food supply in the water column near the habitat-forming species (e.g. corals and sponges) [9]. Considering the repeated observations of exclusive occurrence in a coral habitat [13], [10 present work], adult individuals of *P. bogaraveo* seem to be energetically dependent on the hydrographically mediated food production in such an habitat.

The more abundant occurrence of *H. dactylopterus* in the coral megahabitat is in agreement with a preferential distribution of this fish associated to corals [13], [10]. From the present study, it seems that the medium-small individuals are those preferentially distributed in the coral habitat. Although this fish uses a wide range of habitats tightly associated with the bottom [46]–[49], it is also frequently observed in coral habitats and available photos show solitary individuals resting on the substrate near corals [6], [50], [49], [8], [51], [13], [19]. D'Onghia et al. [10] observed a behavioural pattern of resting on the seabed in different benthic macrohabitats in the SML coral province. *H. dactylopterus* seems to be a typical sit-and-wait ambush predator feeding mainly on benthic crustaceans and fish as well as on plankton organisms [52], [53], [54]. With regard to its planktivorous habit, as suggested for redfish of the genus *Sebastes* [4], hydrographically mediated factors can increase the density of zooplankton in coral habitats. As observed for other rockfish [55], *H. dactylopterus* can be associated with corals for feeding because zooplankton and small shrimps can be more abundant among the colonies. Using longline, Husebø et al. [5] reported greater catches of redfish (*Sebastes marinus*) with larger individuals in coral habitats than in non-coral habitats. Redfish of the genus *Sebastes* also seem to be associated with sponges [56].

*C. conger* had already been collected in the SML coral area using fishing gears [13] and towed cameras, these latter revealing a swimming behaviour near the seabed [10]. This fish is considered a large opportunistic predator living and foraging close to rocky areas where it finds refuge during the day [57], [58]. Sulak et al. [59] report *Conger oceanicus* burrowing into the base of *Lophelia* bushes. The significantly greater abundance recorded both by D'Onghia et al. [13] and during the present study would indicate a preferential distribution of conger eel in structurally complex habitats like those built by deep-sea corals. However, as above mentioned, large individuals of *C. conger*, which roam a vast area searching for food, can also be found in other megahabitats of the SML coral province protected from fishing by their complex topography.

Adult individuals of *P. americanus* are usually solitary swimmers and seem to have a preferential distribution on larger lithoherms and hardgrounds [60] as well as in caves and shipwrecks [61]. The occurrence of the wreckfish in the SML coral province was first recorded by Carbonara et al. [62]. The significant differences between coral and non-coral megahabitats observed in the second survey could be due to the same reasons suggested for the above discussed species.

A remarkable density of juveniles of *E. spinax* in the SML coral province has previously been reported by D'Onghia et al. [13]. The finding of a higher number of medium-small individuals of *E. spinax* in the coral megahabitat during the present study could probably be explained by the fact that corals provide a better refuge for the juveniles of this shark.

The lack of significant differences in the total catches and fish assemblages considering all the sampling stations between coral and non-coral megahabitats, could be due to the fact that both megahabitats investigated include irregular bottoms with different benthic meso-macrohabitats and play the role of attraction-refuge with respect to the northern barren muddy bottoms where fishing occurs. In fact, the occurrence in both coral and non-coral megahabitats of skate species which are rather rare on the Ionian fishing grounds [63] could be explained as a typical refuge effect of the whole SML coral province. It is well known that these cartilagineous fish are particularly vulnerable to overexploitation because of their k-selected life-history strategy [64]. The occurrence of a higher number of large individuals of *G. melastomus* and *C. conger* in the non-coral megahabitat confirms the refuge effect from fishing also for this megahabitat [13]. In the present study, both coral and non-coral megahabitats in the SML coral province are characterized by muddy bottoms interspersed with hard grounds and other complex seafloor morphologies which are less accessible to fishing activities and thereby can provide a natural refuge for mobile fauna, as observed in submarine canyons [65]. Indeed, the north-western non-coral megahabitat is characterized by the presence of a canyon. In addition, *E. spinax*, *G. melastomus*, *C. conger*, *M. merluccius* and some other species collected in the present study are large carnivores and/or scavengers which swim over a vast area searching for food. This could further explain the lack of significant differences between the fish assemblages found in the two megahabitats. Furthermore, the corals in the SML province, as in the rest of Mediterranean, have a patchy distribution, a low density and do not build flourishing reef-like mounds as they do in the Atlantic [66]. Indeed, their occurrence in the Mediterranean appears to be a relict of a much more extensive distribution during the Pleistocene [67]. This could explain the lower impact of corals than that observed in Atlantic [31], [32]. However, a smaller number of longlines and hooks were deployed in the SML coral province with respect to Atlantic studies [31], [32] preventing comparison with these studies and, in our opinion, also making the impact of corals in this Mediterranean coral province rather remarkable.

As reported by other authors [1], [9], [16], the many methodological difficulties in sampling mobile fauna in deep-sea coral habitats leave several open questions on coral-associated fish density and diversity. In spite of such difficulties and although the level of association with corals varies geographically and is influenced by the natural variability of the cold-water coral environment [68], the present results highlight the important role of the whole SML coral province as a refuge area from fishing, irrespective of the megahabitat typology. This is in agreement with the question raised by Auster [15] on the functional equivalence of different complex habitats for fish. In fact, the complex seabed topography in different sites of the SML coral province, due to the presence of hardgrounds, fault scarpments, carbonate mounds, canyons and other seafloor irregularities, makes this area unsuitable for safe commercial fishing. Baker et al. [16] observed that regardless of whether corals play an obligate and functional role for fish, they represent important features within the deep sea and seem to influence fish distribution and abundance. Within the SML coral province, the coral megahabitats show some differences in their fish species distribution. In fact, large specimens of *P. bogaraveo* confirm their close association with the presence of corals [13], [10] and other fish species (mostly *C. conger* and *H. dactylopterus*) show a preferential distribution in the coral habitats than in other habitat typologies. Although habitat use is difficult to demonstrate, future research must address the importance of the SML coral province as an “essential fish habitat” for these fish species. In this respect, as discussed in more detail by D'Onghia et al. [13], in 2006 the General Fisheries Commission for the Mediterranean (GFCM) created the legal category of “Deep-sea Fisheries Restricted Area” for conservation objective, recommending the prohibition of towed gears and dredges in the SML cold-water coral province. From the present study and in agreement with Baker et al. [16], any conservation program aimed at protecting deep-sea ecosystems must protect a wide range of habitats and depths to ensure that a variety of species and assemblages benefit.
